# The high-resolution three-dimensional (3D) chromatin map of the tea plant (*Camellia sinensis*)

**DOI:** 10.1093/hr/uhad179

**Published:** 2023-09-01

**Authors:** Weilong Kong, Jiaxin Yu, Jingjing Yang, Yanbing Zhang, Xingtan Zhang

**Affiliations:** National Key Laboratory for Tropical Crop Breeding, Shenzhen Branch, Guangdong Laboratory for Lingnan Modern Agriculture, Genome Analysis Laboratory of the Ministry of Agriculture, Agricultural Genomics Institute at Shenzhen, Chinese Academy of Agricultural Sciences, Shenzhen, Guangzhou 518120, China; National Key Laboratory for Tropical Crop Breeding, Shenzhen Branch, Guangdong Laboratory for Lingnan Modern Agriculture, Genome Analysis Laboratory of the Ministry of Agriculture, Agricultural Genomics Institute at Shenzhen, Chinese Academy of Agricultural Sciences, Shenzhen, Guangzhou 518120, China; National Key Laboratory for Tropical Crop Breeding, Shenzhen Branch, Guangdong Laboratory for Lingnan Modern Agriculture, Genome Analysis Laboratory of the Ministry of Agriculture, Agricultural Genomics Institute at Shenzhen, Chinese Academy of Agricultural Sciences, Shenzhen, Guangzhou 518120, China; National Key Laboratory for Tropical Crop Breeding, Shenzhen Branch, Guangdong Laboratory for Lingnan Modern Agriculture, Genome Analysis Laboratory of the Ministry of Agriculture, Agricultural Genomics Institute at Shenzhen, Chinese Academy of Agricultural Sciences, Shenzhen, Guangzhou 518120, China; State Key Laboratory of Crop Stress Adaptation and Improvement, School of Life Sciences, Henan University, Kaifeng, Henan 475004, China; Shenzhen Research Institute of Henan University, Shenzhen, Henan 518000, China; National Key Laboratory for Tropical Crop Breeding, Shenzhen Branch, Guangdong Laboratory for Lingnan Modern Agriculture, Genome Analysis Laboratory of the Ministry of Agriculture, Agricultural Genomics Institute at Shenzhen, Chinese Academy of Agricultural Sciences, Shenzhen, Guangzhou 518120, China

Dear Editor,

Tea plant (*Camellia sinensis* (L.) O. *Kuntze*) is one of the world's most important non-alcoholic beverages, with great economic, health, and cultural value. Recently, several genomes of *C. sinensis var. assamica* (CSA) (Yunkang 10), *C. sinensis var. sinensis* (CSS) (Shuchazao, Biyun, Longjing 43, Tieguanyin, Huangdan), and ancient tea plant (DASZ) have been deciphered [1–3], but studies on three-dimensional (3D) chromatin map have not been addressed to date. The 3D chromatin organization functions critically in fundamental biological processes including DNA replication, gene expression regulation, cell division, chromosome recombination, etc. [4] and changes in 3D chromatin organization, accessibility, transcriptional regulatory elements directly affect changes in gene expression, finally leading to differences of important traits [5]. 3D genome of tea plant will provide a multi-layered full view of gene expression regulation modules for complex trait elucidation.

To decipher the high-resolution 3D chromatin map of tea plant, we obtained 622.9 Gb of clean Hi-C reads from six fresh leaf libraries of the Oolong tea variety ‘Tieguanyin’ (TGY), corresponding to 216.63 X sequencing coverages of the TGY genome (Table S1**,** see online supplementary material). The Hi-C map shows a stronger contact signal along the main diagonal and significant separation among chromosomes, suggesting chromatin contact intensity delay as physical distance increases (Fig. 1A). A PCA-based analysis (Materials and Methods, see online supplementary material) of 100-kb resolution Hi-C contact data showed that about 48.87% and 51.13% of the TGY genome, respectively, belonged to 841 A and 826 B compartments containing 27 642 and 17 593 genes, and the percentage of A and B compartments was different among chromosomes (Fig. 1B; Table S2**,** see online supplementary material). This A and B percentage was similar to that of rice (A vs B of 48.31% vs 51.69%) [6], and was different from cotton including *Gossypium arboreum* (A vs B of 54.20% vs 45.80%), *Gossypium hirsutum* (A vs B of 52.90% vs 47.10%), *Gossypium barbadense* (A vs B of 52.40% vs 47.60%) [7], peanut (A vs B of 52.30% vs 47.70%) [5, 7], and pepper (A vs B of 59–65% vs 48.55%) [8], which have a slightly larger A percentage. Similar to other plant 3D genomes [7, 8], A compartments were concentrated near telomeres, while B compartments occupied the intermediate repeat region of the chromosome, such as centromeres (Fig. 1A). These comparison results of percentage and position of A/B compartments among different species imply that the genomic position distribution of A/B compartments may be more conserved than the percentage. Earlier studies have demonstrated that A and B compartments have different genomic, transcriptomic, and epigenomic features, and that these differences are closely related to A/B functional compartmentalization (e.g. transcriptional activation or repression) [5–8]. We therefore analysed the genomic, transcriptomic, and epigenomic features of tea plant A/B compartments using WGBS-seq, H3K27ac Chi-seq, ATAC-seq, and RNA-seq (Table S3**,** see online supplementary material), and noted that A compartments were enriched for genes, CHH DNA methylation, H3K27ac ChIP-seq signals and ATAC-seq signals, which tend to mark active chromatin states (Fig. 1C; Fig. S1, see online supplementary material). But B compartments had higher levels of GC ratio, LTR density, CG and CHG DNA methylation relative to that of A compartments, and these features are closely associated with repressed chromatin. Similar to the findings in the 3D genome of the chili pepper [8], these results suggested that compartments in the tea plant genome also differ in multiple epigenetic modifications, and that such epigenetic differences may govern 3D contact patterns.

**Figure 1:**
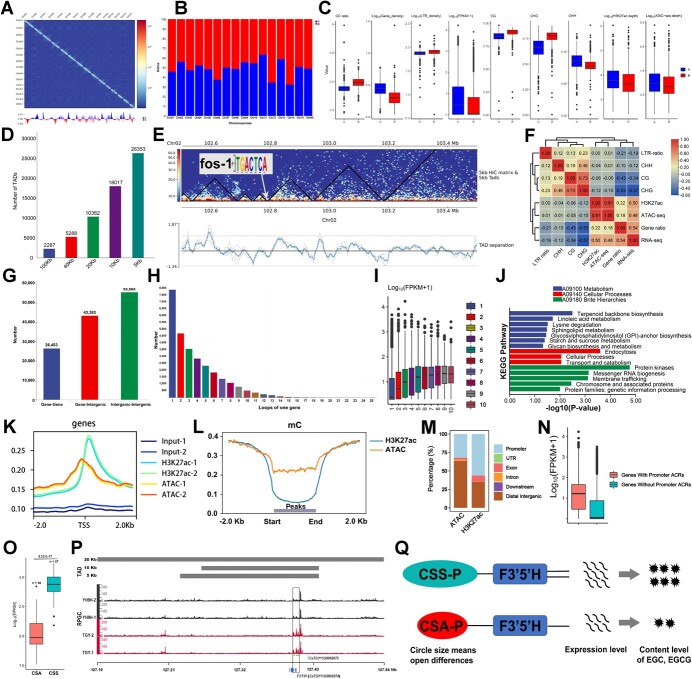
The high-resolution three-dimensional (3D) chromatin map of the tea plant (*Camellia sinensis*). **A** Genome-wide Hi-C interaction matrix and A/B compartments. **B** The proportion of A and B compartments among all chromosomes. **C** The genomic, transcriptomic, and epigenomic features of A and B compartments. **D** Number of topological associated domains (TADs). **E** The motif enrich result of TADs boundary. **F** The Pearson correlation of multiple markers within TADs. **G** Classification of loops. **H** The number of loops linked to one gene. **I** Gene expression levels of genes with different numbers of loops. **J** The KEGG enrichment result of multi-loops regulated genes. **K** Read depth in the 2 Kb upstream and downstream of the transcription start sites (TSSs) of genes. **L** The 5 mC methylation modification levels of the 2 Kb upstream and downstream of H3K27ac and ATAC-seq peaks. **M** The genomic position annotation of ATAC-seq and H3K27ac peaks. **N** Gene expression level of gene with/without promoter accessible chromatin regions (ACRs). **O** Gene expression level of *F3'5'H* gene in *Camellia sinensis var. assamica* (CSA) and *Camellia sinensis var. sinensis* (CSS) accessions. **P** Differential chromatin accessibility from ATAC-seq data around the *F3'5'H* gene between ‘Tieguanyin’ (TGY, CSS) and ‘Yinghong No. 9’ (YH9H, CSA). **Q** The putative regulatory model of the differential accumulation of EGC and EGCG in CSA and CSS accessions.

Topological associated domains (TADs) are of great importance in gene expression regulation, and TAD-like domains are widespread in multiple plants [5, 7, 8]. We respectively obtained 2287, 5288, 10 382, 18 017, and 26 353 TADs at 100 Kb, 40 Kb, 20 Kb, 10 Kb, and 5 Kb resolution by hicFindTADs (Fig. 1D). In 5 Kb-resolution, the length of TADs was centered around 55 kb, and about 70% of TADs contained more than one gene (Figs S2 and S3, see online supplementary material). We found that the boundaries of tea plant TADs were enriched with fos-1 transcription factors (TFs), implying that fos-1 TF may be involved in the formation of TAD structures in tea plant (Fig. 1E). Of course, this hypothesis awaits further functional validation by characterizing the 3D genomic differences between the *fos-1* mutant and WT plants once the tea plant gene editing and transformation technologies are mature. The Pearson correlation result reveals that repressive and active markers are clustered into two independent clusters, respectively (Fig. 1F). Based on a hierarchical clustering of these markers, we gained three major groups of TADs and defined in Cluster 1 (*n* = 12 129) as repressive due to their enrichment for repressive markers and defined Cluster 3 (*n* = 9404) as active because of their high levels of active markers (H3K27ac, ATAC-seq, RNA-seq, and gene density) (Fig. S4, see online supplementary material). Cluster3 has the highest level of active markers (gene density, RNA-seq), Cluster1 has the highest level of three methylation marks, while Cluster 2 (*n* = 4820) had moderate expression levels among three clusters but had the highest levels of H3K27ac and ATAC-seq signals (Fig. S5, see online supplementary material).

At 5Kb resolution, we identified 125 404 loop structures (loops) among all chromosomes by Hiccups CPU mode (Fig. S6, see online supplementary material) [9]. Overall, number of loops decayed with increasing distance, reaching 57.91% for loops within 400 Kb (Fig. S7, see online supplementary material). Based on whether the loop anchors were overlapping with genes, the total loops were subdivided into 55 568 intergenic-intergenic loops, 43 383 gene-intergenic loops, and 26 453 gene–gene loops, implying that most of the loops regulate gene expression through the interaction between long-distance regulatory elements (enriched in intergenic regions) and genes (Fig. 1G). To further characterize the effect of loops on gene expression, we analysed the number of loops associated with one gene and found that the vast majority of genes only participate in a loop structure (Fig. 1H). Compared with genes with only one loop, genes with multiple loops had higher gene expression levels, indicating that the gene expression levels were positively correlated with the number of loops overall (Fig. 1I). A gene with multiple loops implies that the gene may be regulated by multiple remote transcription elements or have transcriptional regulatory relationships with multiple genes, which may explain why these genes have higher expression levels. We therefore performed KEGG enrichment and TF identification on genes (*n* = 1551) with more than 10 loops. KEGG results revealed that these multi-loops regulated genes involved multiple mentalism-, cellular process-, and brite hierachy pathways and also played an essential role in the synthesis of major aroma substances (related to terpenoid backbone biosythesis) (Fig. 1J). Of multi-loops regulated genes, we also identified many TFs, meaning that these TFs may have potential regulatory relationships with multiple genes or transcriptional regulatory elements through loops, and they may play non-negligible roles in the growth and development of tea plant (Fig. S8, see online supplementary material).

In our 3D genome study, we found that the vast majority of loop anchors fall in non-coding intergenic regions (Fig. 1G). Due to non-coding intergenic regions containing rich transcriptional regulatory elements [8, 10], it is important to analyse genome-wide transcriptional regulatory elements to understand loops-mediated transcriptional regulation. We therefore performed ATAC-seq and H3K27ac Chip-seq of the same tea plant accessions (the young leaf of ‘TGY’) and found that ATAC-seq and H3K27ac Chip-seq reads were enriched at the transcription start sites (TSSs) of genes (Fig. 1K; Fig. S9, see online supplementary material). We respectively identified 33 727 and 18 602 reproducible peaks (Figs S10–S12, see online supplementary material) from ATAC-seq by MACS2 (2.2.7.1) with qvalue ≤0.05 and H3K27ac Chip-seq and the H3K27ac peaks were longer in length than those of ATAC-seq peaks (Fig. S13, see online supplementary material). We found that the body of peaks had lower methylation levels than the upstream and downstream of H3K27ac and ATAC-seq peaks, consistent with their characteristics as active marks (Fig. 1L). Because ATAC-seq peaks representing genome accessible chromatin regions (ACRs) can contain promoters and enhancers [7, 8], we thus divided ATAC-seq peaks into 10 003 promoter ACRs (located 3000 bp upstream of genes, defined as promoter elements in this study), 21 880 distal intergenic ACRs, and other ACRs based on the genomic positions of peaks (Fig. 1M). Promoter ACRs were significantly enriched in H3K27ac Chip-seq signals relative to distal intergenic ACRs (Fig. S14, see online supplementary material) and promoter ACRs-related genes had higher gene expression levels (Fig. 1N). This result is consistent with the findings of promoter ACRs in cotton [11], highlighting the positive role of promoter ACRs in regulating gene expression. We further defined H3K27ac peaks (*n* = 6510) that fell to distal intergenic as enhancers and defined enhancers overlapping ATAC-seq peaks as active enhancers (*n* = 1434, 22% of all enhancers, Fig. S15, see online supplementary material). We further integrated promoters and enhancers into loops in tea plant and identified 3886 loop single anchors containing enhancers and 5868 genes regulated by enhancers through loops (Table S4, see online supplementary material). The KEGG enrichment results revealed that these enhancer-affected genes were widely involved in a variety of tea plant life activities and were closely related to the survival and the synthesis of various secondary metabolites in tea plant (Fig. S16, see online supplementary material). To investigate the roles of sequence variants in enhancers and promoters on secondary metabolic synthesis regulation in tea plant, we analysed the distribution of our previously identified secondary metabolites significantly associated SNPs [12] in enhancers and promoters. We found 202 SNPs in 170 promoters associated with 112 secondary metabolites (Table S5, see online supplementary material) and 247 SNPs in 181 enhancers associated with 116 secondary metabolites (Table S6, see online supplementary material), and the number of metabolite-associated SNPs within promoters (3.41 SNPs/Kb) and enhancers (2.40 SNPs/Kb) was much higher than the genome-wide average (0.15 SNPs/Kb), suggesting that genetic variations at promoters and enhancers is important for the differences in the content of secondary metabolites in tea plant population. This result not only highlighted metabolite-associated enhancers and promoters in tea plant, but also deepened our understanding of the variations in non-coding regions regulating trait differences.

Studies have shown that chromatin accessibility differences within TADs can influence gene expression by affecting the binding of TFs and other regulatory proteins to the DNA [5, 13]. Our previous study has showed that the *F3'5'H* gene had a higher expression level in pure CSS than that in pure CSA, which contributed to the accumulation of more EGC and EGCG in CSS (Fig. 1O) [12]. We also found four SNPs in the upstream 2 K of the *F3'5'H* gene that are fully differentiated between CSS and CSA (Fst = 1) [12], but the effect of these SNP variants on TAD structure and chromatin accessibility is not clear. The results of TADs identification and chromatin accessibility analysis in this study provide an opportunity to resolve this important issue. In 5 Kb- 20 Kb resolution, *F3'5'H* gene can be contained in a TAD completely (Fig. 1P). In 5 Kb-resolution TAD region, we identified two differential enriched peaks from ATAC-seq data in the *F3'5'H* gene body and in upstream region between TGY (CSS) and Yinghong No. 9 (YH9H, CSA). These two differential peaks resulted in higher chromatin accessibility in TGY compared to that in YH9H, which in turn led to higher levels of gene expression level of *F3'5'H* gene and higher accumulation of EGC and EGCG in TGY than that in YH9H (Fig. 1Q).

In summary, we integrated genome, RNA-seq, ATAC-seq, H3K27ac-based Chip-seq, and WGBs-seq data to create a high-resolution 3D chromatin map containing promoters and enhancers of tea plant. Also, we characterized genomic, transcriptomic, and epigenomic features of three-level 3D structural units and preliminary explored the effects of structural variations in TADs, promoters, and enhancers on gene expression, or/and flavor metabolite accumulation in tea plant population. These results will provide important references for future studies on the mechanisms of gene expression regulation of complex traits.

## Acknowledgements

This study was funded by Shenzhen Science and Technology Program (Grant No. RCYX20210706092103024) and Key-Area Research and Development Program of Guangdong Province (2020B020220004).

## Author contributions

X.Z. and W.K. conceived the ideas for this paper. W.K. performed all of the experiments, analysed the data, prepared the figures and tables, and wrote the paper. J.Y. conducted part of the experiments. J.Y. completed the visualization of some results. All authors read and approved the final manuscript.

## Data availability

All sequencing datasets have been deposited in the National Genomics Data Center (NGDC) under accession number PRJCA017759 (Hi-C data, ATAC-seq, and H3K27ac Chip-seq) and PRJCA014523 (RNA-seq and WGBs-seq).

## Conflict of interest statement

The authors declare that they have no competing interests.

## Supplementary data

Supplementary data is available at *Horticulture Research* online.

## Supplementary Material

Web_Material_uhad179
