# Supplementary material for: The high-resolution three-dimensional (3D) chromatin map of the tea plant (Camellia sinensis)
Source: Hortic Res. 2023 Sep 1;10(10):uhad179. doi: 10.1093/hr/uhad179 (PMC10599236; doi:10.1093/hr/uhad179)
Supplement: Web_Material_uhad179 [file web_material_uhad179.zip › Figure S.docx]

# Supplemental Information


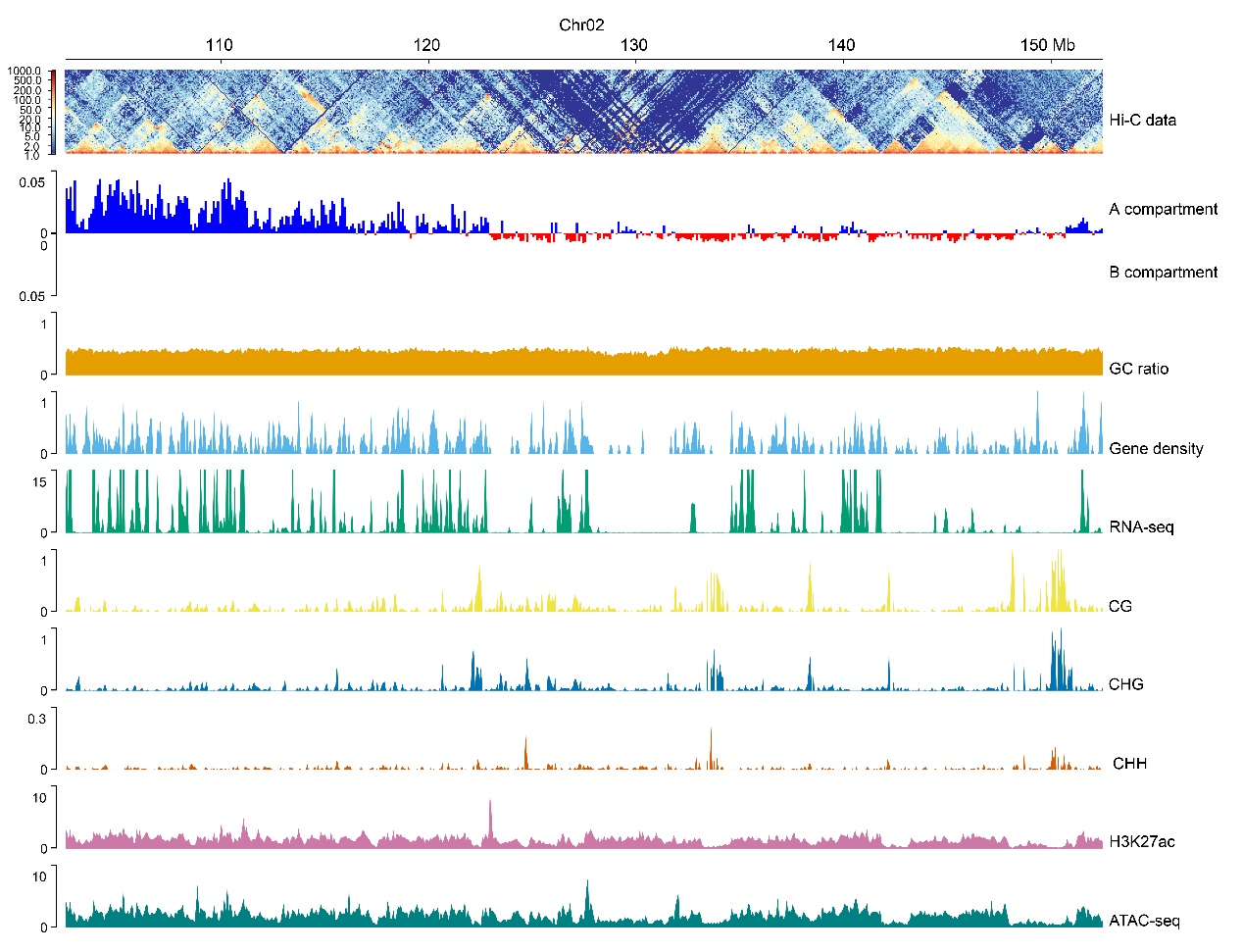


**Figure S1.** A representative example of A/B compartments and sub-compartments in a 50 Mb region on chromosome 2. The below panels show genomic and epigenomic feature profiles.


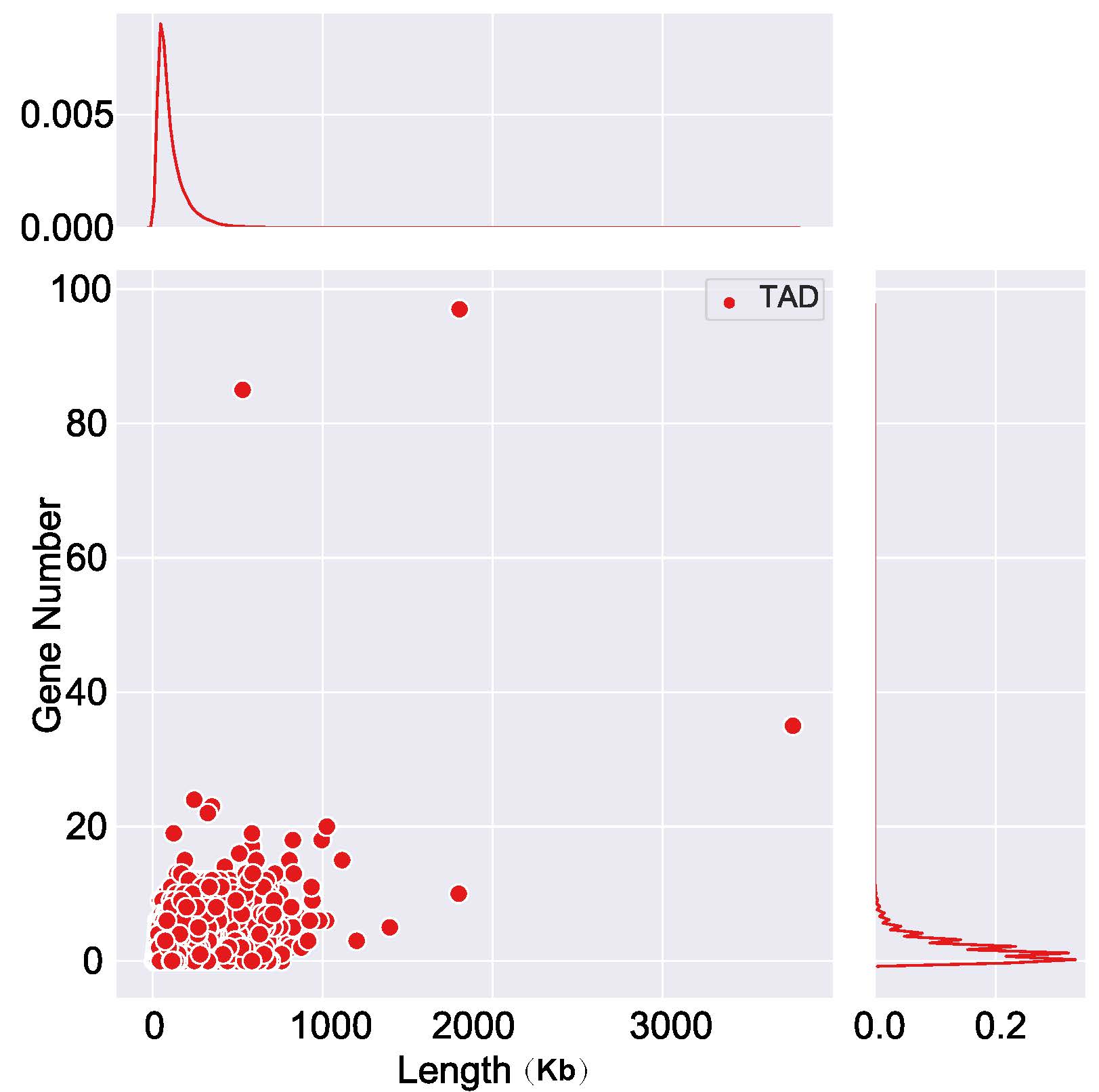


**Figure S2.** The gene number and length of 5Kb-resolution TADs.


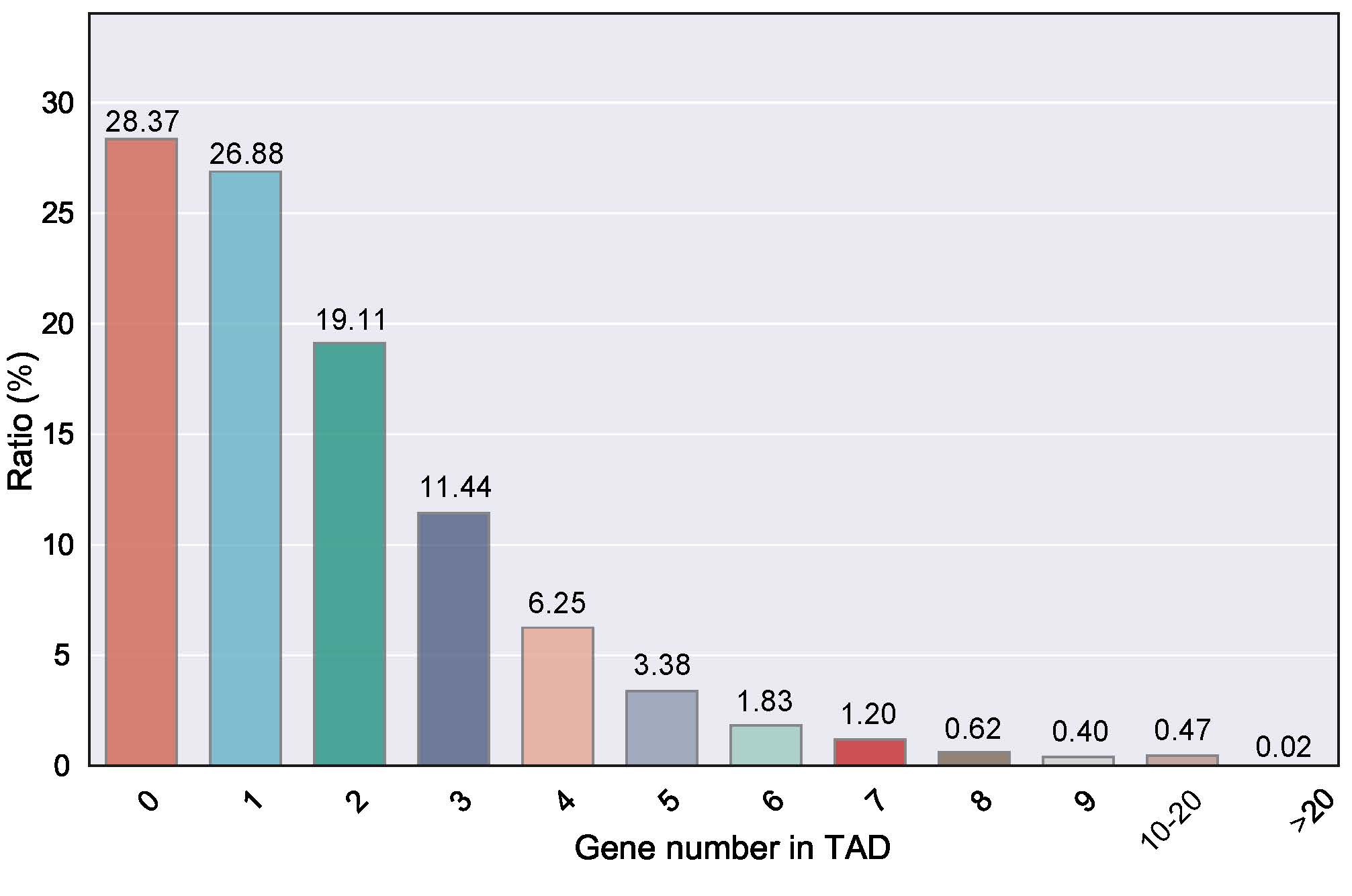


**Figure S3.** The gene number in TADs.


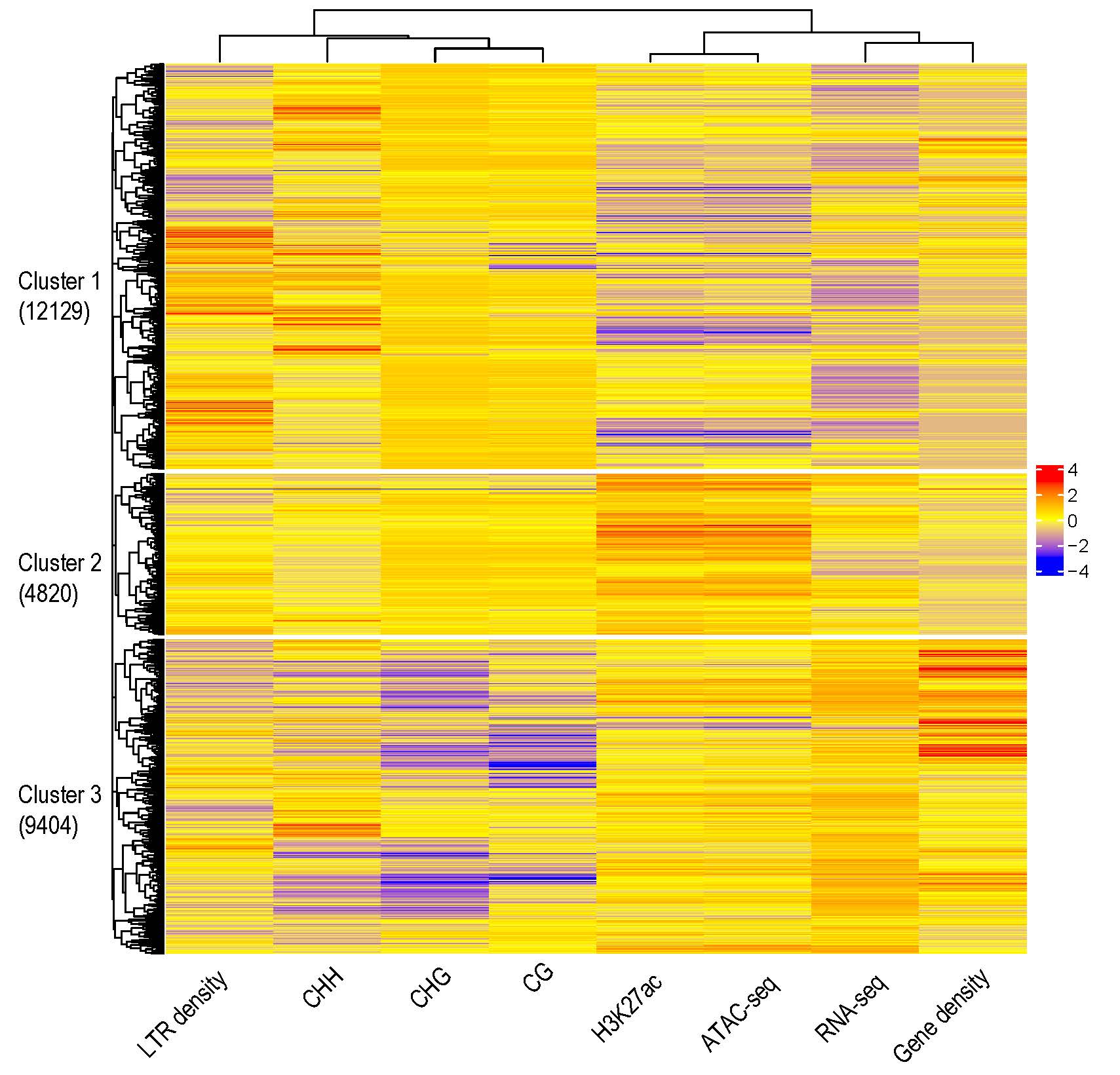


**Figure S4.** A hierarchical clustering of a set of genomic and epigenomic markers in TADs.


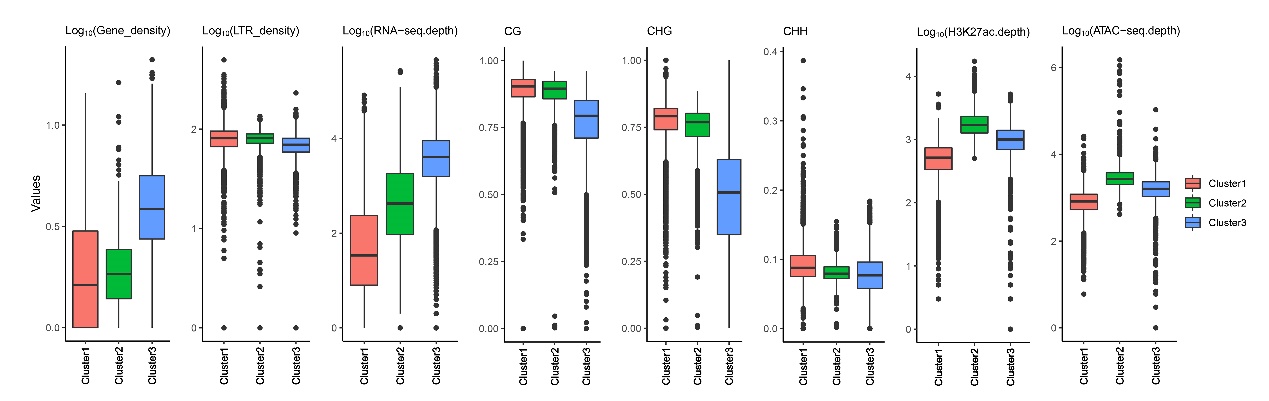


**Figure S5.** Multiple genomic, expression, and epigenomic markers of three TAD clusters.


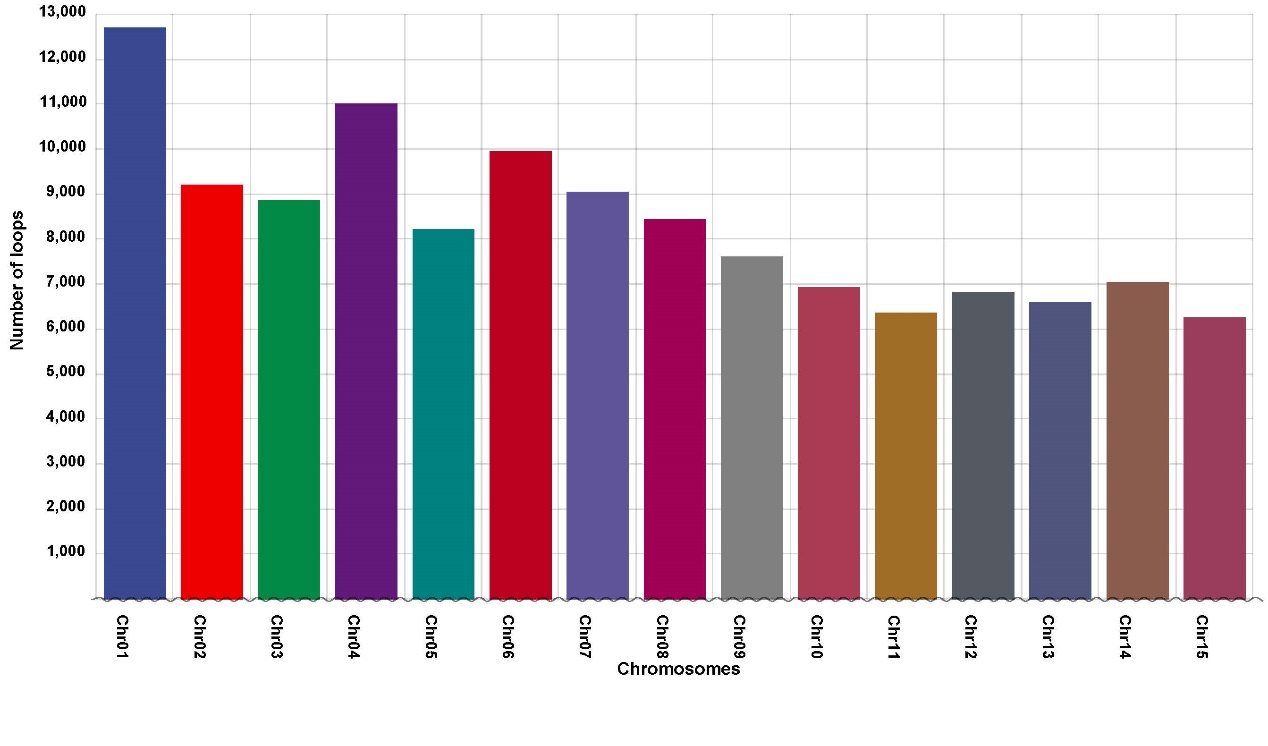


**Figure S6.** Number of loops among 15 chromosomes.


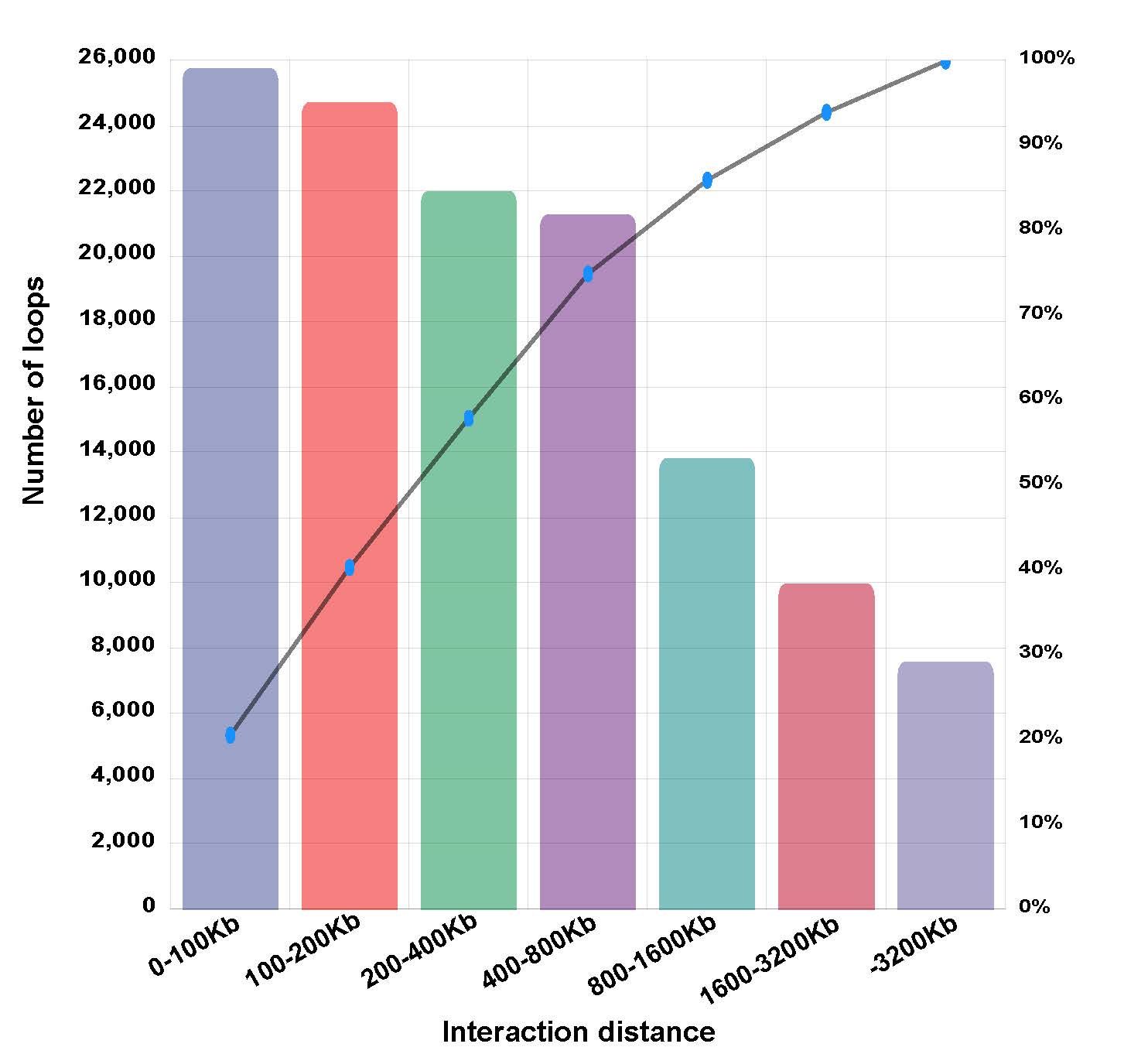


**Figure S7.** Number of loops in different distance.

**
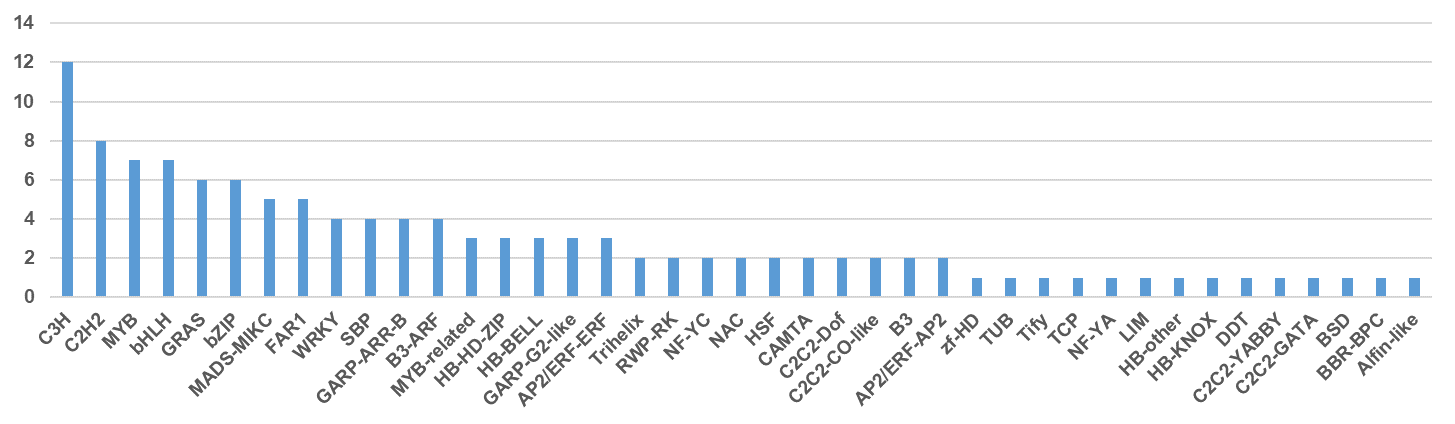
**

**Figure S8.** Transcription factor identification of multi-loops regulated genes.


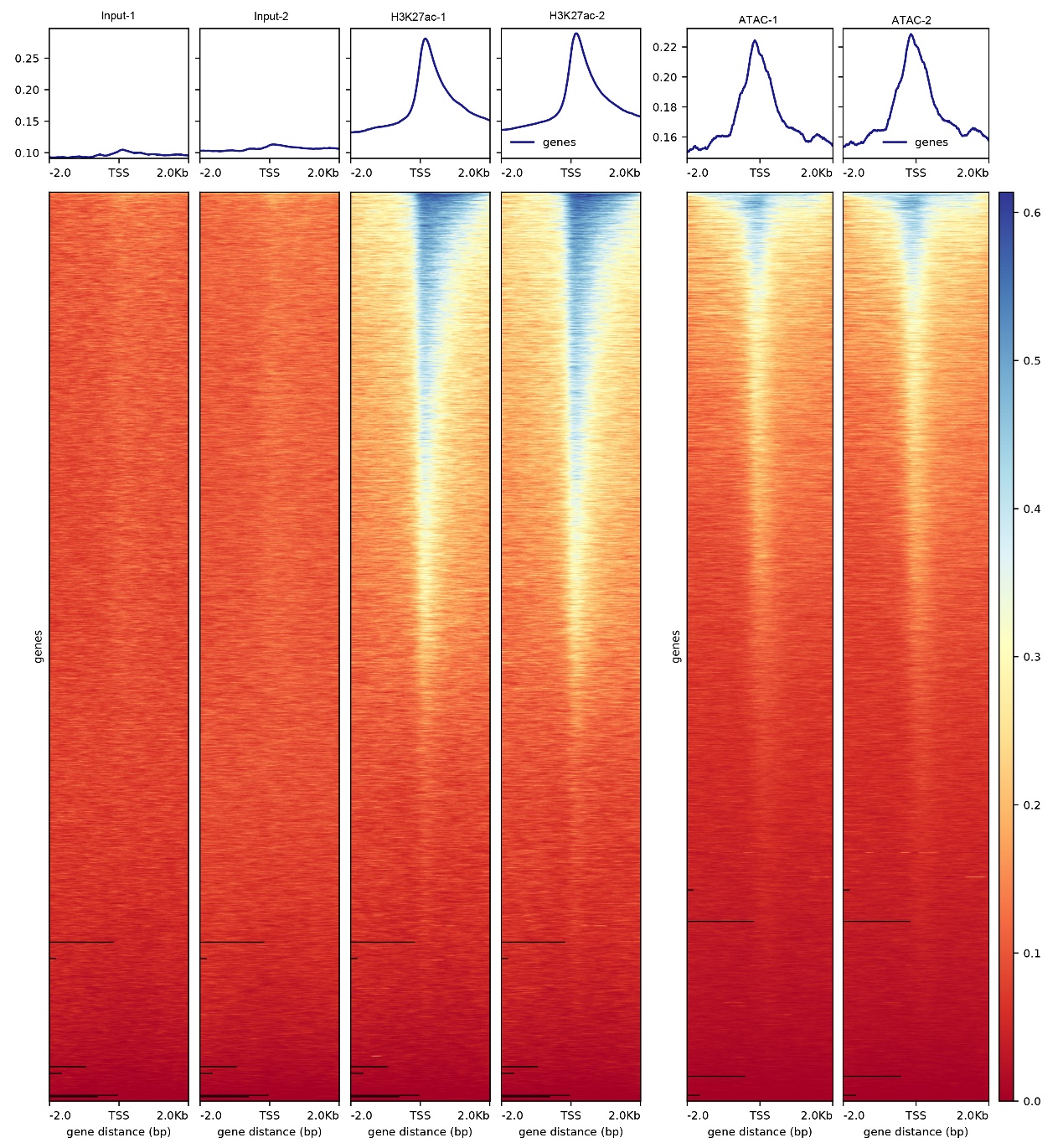


**Figure S9.** Read depth of ATAC-seq and H3K27ac Chip-seq on both sides of the transcription start site (TSS) of genes.

**Figure S10.** Number of ATAC-seq and H3K27ac peaks.


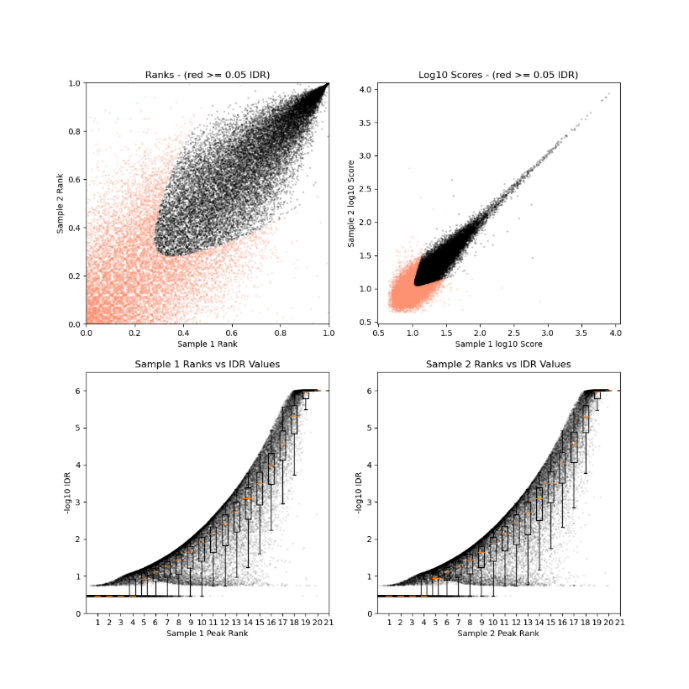


**Figure S11.** Repeatability of two ATAC-seq biological replicates.


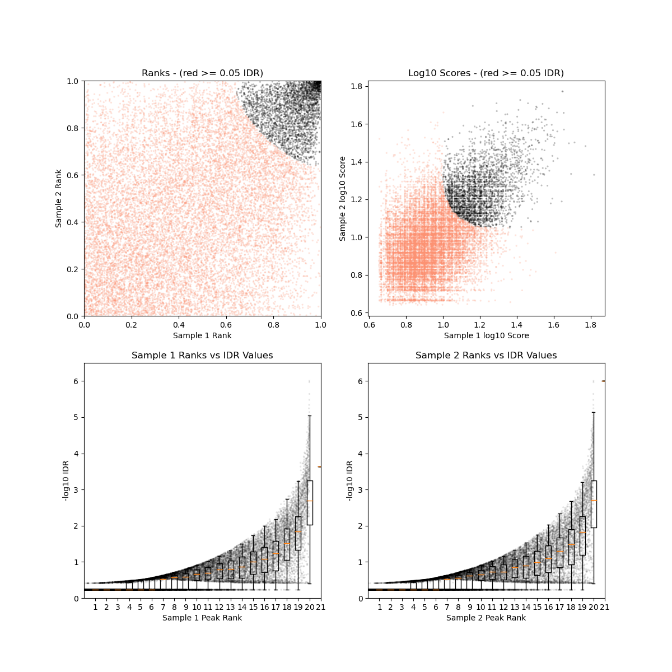


**Figure S12.** Repeatability of two H3K27ac Chip-seq biological replicates.


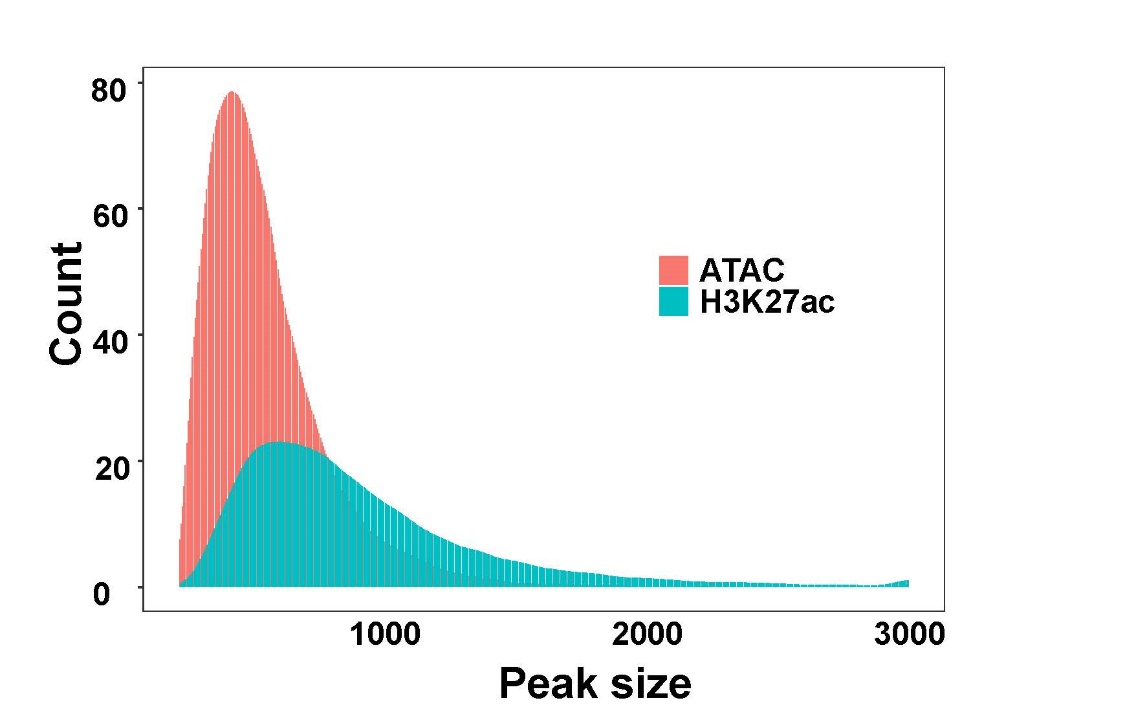


**Figure S13.** Count and size of ATAC-seq and H3K27ac peaks.


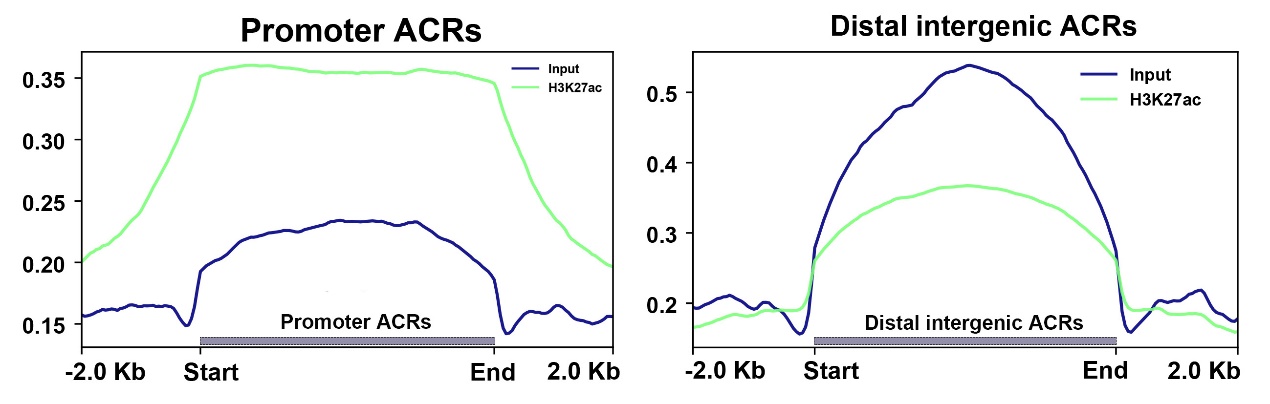


**Figure S14.** The H3K27ac signals in promoter accessible chromatin regions (ACRs) and distal intergenic ACRs.


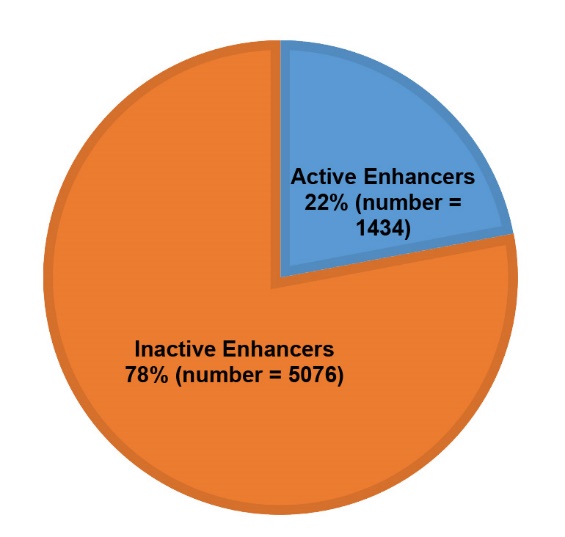


**Figure S15.** Percentage and number of active and inactive enhancers.


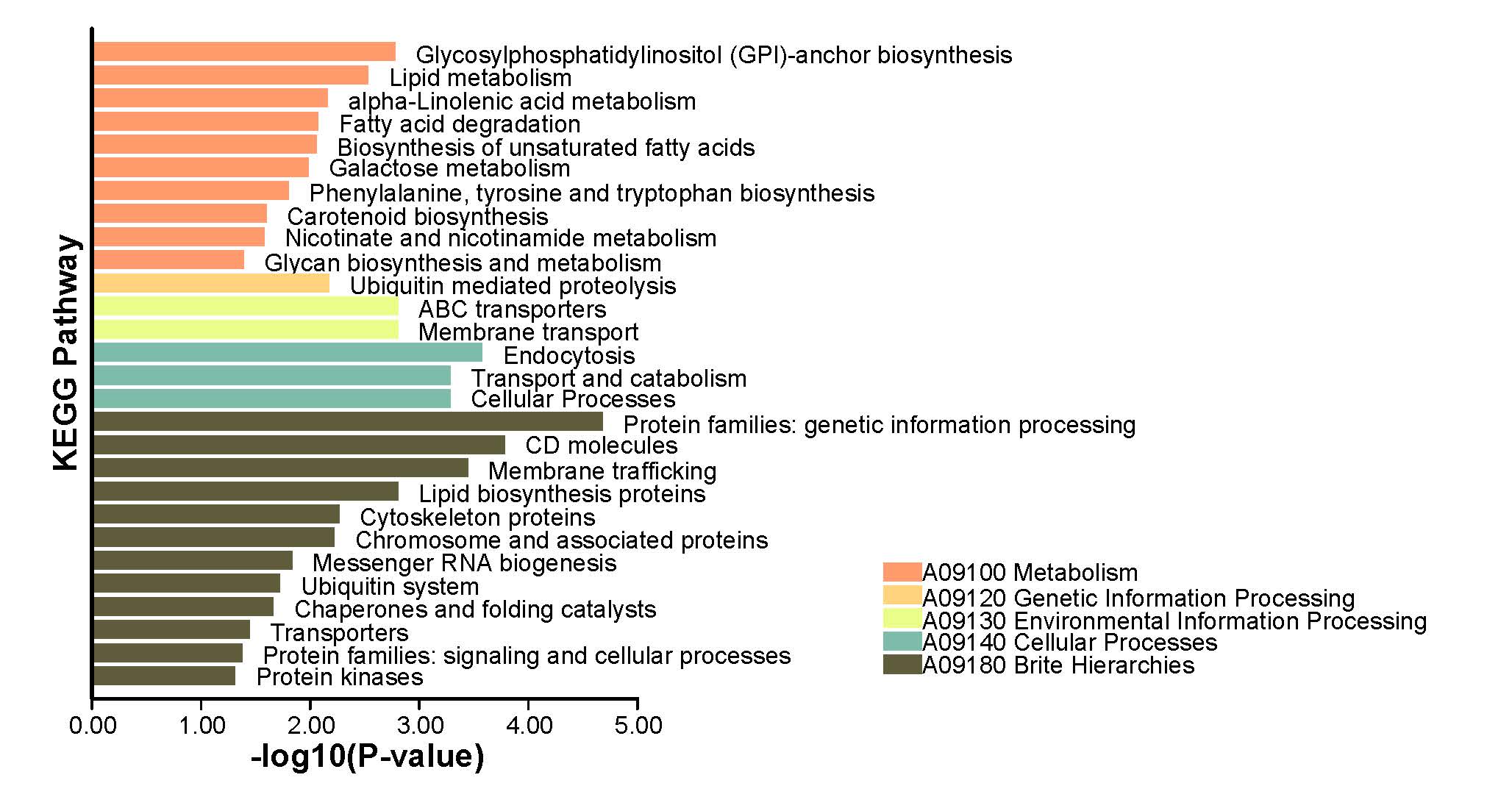


**Figure S16. KEGG enrichment of enhancer-affected genes.**
