# Supplementary material for: The high-resolution three-dimensional (3D) chromatin map of the tea plant (Camellia sinensis)
Source: Hortic Res. 2023 Sep 1;10(10):uhad179. doi: 10.1093/hr/uhad179 (PMC10599236; doi:10.1093/hr/uhad179)
Supplement: Web_Material_uhad179 [file web_material_uhad179.zip › Materials and Methods.docx]

# Supplemental Information

# Materials and Methods

## Plant material

Tea leaves were collected from fresh leave of the Oolong tea variety “Tieguanyin” (TGY, *C. sinensis* var. *sinensis*).

**Hi-C experiment, sequencing, and data processing**

Hi-C libraries of fresh leave of TGY were constructed according to the protocol established by Rao et. al. (2014) [1] and DNA restriction enzyme is HindIII. Sequencing was performed (150 bp paired-end) on the Illumina NovaSeq (2 cells) platform and MGISEQ-T7 (4 cells) platform. We mapped clean Hi-C reads to the TGY reference genome [2] using HiC-Pro [3] and produced Hi-C interactions matrices at 5kb, 10kb, 20kb, 40kb, 100kb resolution respectively. We further used iced [4] to correct the genome-wide interaction matrix at different resolutions. Genome-wide HiC interaction matrix at the iced normalized 100 kb resolution was used for the identification of A and B compartments. The interaction matrix was first split into chromosomes and the Z-score transformation was performed by matrix2loess.pl in Cworld-dekker (https://github.com/dekkerlab/cworld-dekker) for interaction matrix of each chromosome, and the corrected matrix was subsequently analyzed using matrix2EigenVectors.py for principal component analysis (PCA). The PCA1 region with more transcripts is the A compartment and vice versa for the B compartment.

The corrected HiC interaction matrix with different resolutions was used for the identification of TADs. We first converted the interaction matrix to .h5 format using hicConvertFormat in hicExplorer (version 3.7.1) [5], and then used hicFindTADs in hicExplorer to identify TADs based on the TAD-separation score with parameters: --correctForMultipleTesting fdr --minDepth 60kb --maxDepth 100kb --step 5kb --thresholdComparisons 0.05 --delta 0.01. In addition, TAD boundary and TAD (as control) sequences were submitted to the AME V 5.5.3 website (https://meme-suite.org/meme/tools/ame) for standard (non-local) motif enrichment analysis with default parameters.

We used the Hiccups method in Juicer 1.6 [6] to identify the loops structure from the corrected 5kb resolution HiC interaction matrix, and select the "--CPU" parameter to enable the hiccups method to run in CPU mode. KEGG annotation (default parameters) and KEGG enrichment (P value < 0.05) were performed by EggNOG-mapper (<http://eggnog5.embl.de/#/app/home>) and TBtools v1.120 [7]. In addition, we identified transcription factors in multi-loops regulated genes using iTAK software [8] with default parameters.

## Transcriptome sequencing and data analysis

We extracted all RNAs of tea plant samples using the Tiangen total RNA Extraction Kit (product id: DP441, Tiangen, Beijing, China), and then conducted mRNA enrichment using magnetic beads with Oligo (dT). All cDNAs were reverse-transcribed and cDNA libraries were constructed using the QiaQuick PCR kit (Qiagen, Venlo, The Netherlands), and all cDNA sequencing libraries were sequenced on the Illumina HiSeq2500 platform using the paired-end 150 bp method. The raw data were filtered using fastp with default parameters [9]. In this study, our recently published TGY high-quality genome [10] was used as the reference genome for read mapping using Hisat 2.2.1 [11]. The reads were quantified using featureCounts [12].

## Genome-wide DNA 5mC methylation sequencing and data analysis

We extracted the total DNAs of all samples using a modified CTAB method [13] and analyzed DNA extraction quality using 1% agarose gel electrophoresis, OD260/280 ratios, DNA concentration of all samples with Nanodrop and Qubit 2.0. The samples that met the quality control criteria were selected for library construction. The lambda DNA was mixed into the tested DNA samples to assess the quality of bisulfite conversion rate. The genomic DNA was randomly cut into to 200-400 bp DNA fragments using Covaris M220, and the interrupted DNA fragments were end-repaired, A-tailed, and ligated to the sequencing linker in which all cytosines were methylated. After bisulfite treatment using EZ DNA Methylation Gold Kit (Zymo Research, Orange, USA), the unmethylated-C nucleobase in DNA libraries becomes T nucleobase after PCR amplification, but the methylated-C nucleobase remains unchanged. Finally, PCR amplification was conducted to yield the WGB sequencing libraries. All WGB sequencing libraries were sequenced by the paired-end 150 bp method on the Illumina HiSeq sequencing platform.

The WGB raw data were filtered using fastp [9] with parameters: “--cut_front/tail/right_window_size 4 and --cut_front/tail/right _mean_quality 20; --detect_adapter_for_pe -q 15 -u 40 -e 20 -n 5 -l 30 -p -P 20 -w 4”. BatMeth2 pipel [14] (with default parameters) was used to map the filtered WGB reads onto the TGY genome [10] and completed the identification of 5mC methylation sites and calculation of methylation levels of all 5mC methylation sites. The bam files were converted into bw files by batmeth2_to_bigwig.py in BatMeth2 [14] and the upstream and downstream methylation levels of peaks were calculated by methyGff in BatMeth2.

## ATAC-seq, H3K27ac ChIP-seq and data processing

ATAC-seq, H3K27ac (product id: ab4729, abcam, Cambridge, UK) ChIP-seq experiments, and library preparation protocols were conducted as the method described in a previous paper [15] and all qualified libraries were sequenced by the paired-end 150 bp method on the Illumina HiSeq sequencing platform. Two replicates were performed for each ATAC-seq and H3K27ac ChIP-seq. To measure the genome-wide signal profiles of the ATAC-seq or H3K27ac ChIP-seq, raw reads were filtered using fastp [9] with default parameters and were then mapped to the TGY genome using bowtie2 Version 2.5.0 with default parameters [16]. We further filter out low quality mapping reads (Q10) in bam files using samtools Version 1.17 [17] and filter out duplicate mapping reads in bam files by picard Version 2.25.5 with default parameters. H3K27ac and ATAC peaks were called using MACS2 (2.2.7.1) [18] with parameters: -t H3K27ac.bam -c input.bam -f BAMPE -B –SPMR -q 0.05 -g hs --keep-dup all and with parameters: -t ATAC.bam -f BAMPE -B –SPMR -q 0.05 -g hs --keep-dup all, respectively. The ChIP-seq and ATAC-seq signal intensity of a genomic bin was calculated using the bamCoverage tool from DeepTools (3.3.0) [19] with the read coverage normalized in RPGC method. The ChIP-seq or ATAC-seq overlapped peaks from two single replicates were obtained by idr Version 2.0.3 (https://github.com/nboley/idr).

## References

1. Rao SSP, Huntley MH, Durand NC, Stamenova EK, Bochkov ID, Robinson JT, Sanborn AL, Machol I, Omer AD, Lander ES *et al*. A 3D map of the human genome at kilobase resolution reveals principles of chromatin looping. *Cell*. 2014, **159**(7);1665-1680.

2. Zhang XT, Chen S, Shi LQ, Gong DP, Zhang SC, Zhao Q, Zhan DL, Vasseur L, Wang YB, Yu JX *et al*. Haplotype-resolved genome assembly provides insights into evolutionary history of the tea plant Camellia sinensis. *Nature Genetics*. 2021, **53**(8);1250-+.

3. Servant N, Varoquaux N, Lajoie BR, Viara E, Chen CJ, Vert JP, Heard E, Dekker J, Barillot E. HiC-Pro: an optimized and flexible pipeline for Hi-C data processing. *Genome Biology*. 2015, **16**.

4. Imakaev M, Fudenberg G, McCord RP, Naumova N, Goloborodko A, Lajoie BR, Dekker J, Mirny LA. Iterative correction of Hi-C data reveals hallmarks of chromosome organization. *Nature Methods*. 2012, **9**(10);999-+.

5. Ramirez F, Bhardwaj V, Arrigoni L, Lam KC, Gruning BA, Villaveces J, Habermann B, Akhtar A, Manke T. High-resolution TADs reveal DNA sequences underlying genome organization in flies. *Nature Communications*. 2018, **9**.

6. Durand NC, Shamim MS, Machol I, Rao SSP, Huntley MH, Lander ES, Aiden EL. Juicer provides a one-click system for analyzing loop-resolution Hi-C experiments. *Cell Systems*. 2016, **3**(1);95-98.

7. Chen CJ, Chen H, Zhang Y, Thomas HR, Frank MH, He YH, Xia R. TBtools: An integrative toolkit developed for interactive analyses of big biological data. *Molecular Plant*. 2020, **13**(8);1194-1202.

8. Jin JP, Tian F, Yang DC, Meng YQ, Kong L, Luo JC, Gao G. PlantTFDB 4.0: toward a central hub for transcription factors and regulatory interactions in plants. *Nucleic Acids Research*. 2017, **45**(D1);D1040-D1045.

9. Chen SF, Zhou YQ, Chen YR, Gu J. fastp: an ultra-fast all-in-one FASTQ preprocessor. *Bioinformatics*. 2018, **34**(17);884-890.

10. Zhang XT, Chen S, Shi LQ, Gong DP, Zhang SC, Zhao Q, Zhan DL, Vasseur L, Wang YB, Yu JX *et al*. Haplotype-resolved genome assembly provides insights into evolutionary history of the tea plant *Camellia sinensis*. *Nature Genetics*. 2021, **53**(8);1250-1259.

11. Kim D, Landmead B, Salzberg SL. HISAT: a fast spliced aligner with low memory requirements. *Nature Methods*. 2015, **12**(4);357-U121.

12. Liao Y, Smyth GK, Shi W. featureCounts: an efficient general purpose program for assigning sequence reads to genomic features. *Bioinformatics*. 2014, **30**(7);923-930.

13. Attitalla IH. Modified CTAB method for high quality genomic DNA extraction from medicinal plants. *Pakistan Journal of Biological Sciences* 2011, **14**(21);998-999.

14. Zhou QW, Lim JQ, Sung WK, Li GL. An integrated package for bisulfite DNA methylation data analysis with Indel-sensitive mapping. *Bmc Bioinformatics*. 2019, **20**;47.

15. Liao Y, Wang JT, Zhu ZS, Liu YL, Chen JF, Zhou YF, Liu F, Lei JJ, Gaut BS, Cao BH *et al*. The 3D architecture of the pepper genome and its relationship to function and evolution. *Nature Communications*. 2022, **13**(1).

16. Langmead B, Salzberg SL. Fast gapped-read alignment with Bowtie 2. *Nature Methods*. 2012, **9**(4);357-U354.

17. Danecek P, Bonfield JK, Liddle J, Marshall J, Ohan V, Pollard MO, Whitwham A, Keane T, McCarthy SA, Davies RM *et al*. Twelve years of SAMtools and BCFtools. *Gigascience*. 2021, **10**(2).

18. Zhang Y, Liu T, Meyer CA, Eeckhoute J, Johnson DS, Bernstein BE, Nussbaum C, Myers RM, Brown M, Li W *et al*. Model-based analysis of ChIP-Seq (MACS). *Genome Biology*. 2008, **9**(9).

19. Ramirez F, Ryan DP, Gruning B, Bhardwaj V, Kilpert F, Richter AS, Heyne S, Dundar F, Manke T. deepTools2: a next generation web server for deep-sequencing data analysis. *Nucleic Acids Research*. 2016, **44**(W1);W160-W165.
